# Awareness of dehydration state and fluid intake practice among adults population in the Jazan Region of Saudi Arabia, 2019

**DOI:** 10.1017/jns.2021.81

**Published:** 2021-10-04

**Authors:** Osama B. Albasheer, Abdullah Hakami, Abdullah A. Al Faqih, Ibrahim Akkam, Safwan K. Soraihy, Ahmad Mathary, Ali A. Alharbi, Mohammed Yaqoub, Majed A. Alotayfi

**Affiliations:** 1 Department of Family and Community Medicine, Faculty of Medicine, Jazan University, Jazan, Kingdom of Saudi Arabia; 2 Faculty of Medicine, Jazan University, Jazan, Kingdom of Saudi Arabia

**Keywords:** Dehydration state, Dry skin, Prevention, Renal stones, Thirst, Water intake

## Abstract

Despite the hot climate and high humidity in the Jazan Region of Saudi Arabia, which increases risk for dehydration, no previous studies have assessed awareness of dehydration and fluid intake practice among adults in this region. Therefore, the aim of this cross-sectional study was to determine awareness of the dehydration state and fluid intake practices among 440 adults in the Jazan Region of Saudi Arabia. Out of the total, 51⋅8 % were male and 48⋅2 % were females. Good knowledge of dehydration definition and prevention and recommended minimum water intake was observed in 98, 95 and 75 % of the participants, respectively. Fifty-nine percent of the participants met the minimum daily requirement of 3 l or more per day. The age (95 % CI 1⋅003, 1⋅017, *P* value = 0⋅006), diabetes (95 % CI 1⋅028, 1⋅459, *P* value = 0⋅023) and prior hospitalisation due to dehydration (95 % CI 1⋅010, 1⋅378, *P* value = 0⋅037) were associated with higher water intake. Additional glasses of coffee (95 % CI 1⋅02, 1⋅115, *P* value = 0⋅004) and juice (95 % CI 1⋅039, 1⋅098, *P* value < 0⋅001) were associated with more water intake. The participants exhibited good knowledge of dehydration definition, symptoms and consequences. Intake of fluids such as ‘juice and coffee’ enhances more water intake. Although two-thirds of the participants met the recommended daily water intake, still one-third of them did not meet this level. Innovative approaches to enhance healthy drinking are warranted and may include partnering with patients to take an active role in hydration monitoring and increasing communication with the different healthcare providers.

## Introduction

Water plays a fundamental role in the biological structure and function of all living cells^(1)^. Water regulates body temperature, protects body organs and tissues, helps dissolve minerals and nutrients to make them accessible and helps regulate acid-base balance. In a healthy adult, with an average 70 kg, water accounts for 60 % of body weight and the body requires 2–3⋅5 l per day of total water intake^(2)^. Water inputs composed of three major sources: major one result when drinking water and liquid with high water contents, second the water we eat come various food with a large amount of water content and the third source produce from the oxidation of macronutrients (endogenous or metabolic water)^(3)^. A person is considered dehydrated if they lose as little as 3 % of their body weight from water depletion^(2)^. Insufficient water intake with loss of body mass is associated with poor memory and attention^(4)^.

Imbalance between water intake and loss causes a state of dehydration, which is associated with morbidity and mortality particularly in older adults^(5,6)^. The risk of developing constipation, dental carries urinary tract infections and renal stones increases due to dehydration^(7,8)^. The prevalence of dehydration varies with age (16–21 %) and the signs and symptoms include thirst, mouth dryness, decrease in urination or darkening of urine. Rapid weight loss (over 10 % of body weight) is considered as severe dehydration^(9)^.

Awareness of dehydration and fluid intake was found unsatisfactory in cross-sectional surveys among adults in thirteen countries worldwide^(10)^. A study done in China revealed that 28⋅4 % of people under the study were unaware of minimum daily water intake and 48⋅3 % drink water only when they feel thirsty^(11)^. Another study done in Australia reported that 82 % of the population failed to achieve adequate daily water intake^(12)^.

Public awareness of dehydration is not widely assessed in Saudi Arabia. A previous study among people living in Riyadh, Saudi Arabia, to assess awareness of dehydration and fluid intake, displayed good participants’ knowledge of the common symptoms of dehydration, however knowledge was lacking for uncommon symptoms and serious consequences of dehydration^(13)^. Awareness of dehydration and fluid intake in other areas of Saudi Arabia, particularly those with climates that are conducive to dehydration, are needed.

Jazan Region is a subtropical and coastal area that is known of having hot climate and high temperature and humidity for most of the year, with a lack of natural thermal springs, which gets people dehydrated easily. Therefore, the aim of the present study was to determine the knowledge of dehydration state and fluid intake practice among adults of the Saudi Arabian population in the Jazan Region.

## Methods

### Study setting

The present study was conducted among adults living in the Jazan Region. Jazan is the capital of Jazan Region. It is located in the southwest corner of Saudi Arabia and the estimated population, in 2017, was 1⋅6 million^(14)^.

### Ethics approval and consent to participate

Bioethics standards of the Kingdom of Saudi Arabia were considered while conducting this study. Participants were informed that they had the right to withdraw from the study at any time, their information would be kept anonymous and the data collected would only be used for scientific purposes. Written informed consent was obtained from each participant after explaining the purpose of the study. Also, permission was obtained from the administration of all colleges participated in the study before data collection. Finally, ethical approval for this study was provided by the Standing Committee for Scientific Research Ethics-Jazan University (Ref. no: REC41/6/154).

### Study design and population

A cross-sectional, descriptive, study was conducted to gather information related to the awareness of dehydration and fluid intake among adults living in the Jazan Region of Saudi Arabia.

A multi-stage random sampling method was used for conducting this study. A total of 440 participants were enrolled using the Epi Info program formula, used for sample size calculation. Given the unavailability of previous studies in the Jazan Region, we assumed that 50 % of the population were aware of dehydration status. Therefore, calculation of the sample size was managed on the presumption that the non-response rate of 10 %, the confidence level (95 %), the margin of error not more than (5 %) and the total awareness of (50 %). The value of 95 % CI from the normal distribution is 1⋅96 and for practical reasons we rounded it to be 2, so the estimated sample size increased to 440 participants. There were 179 primary health care (PHC) centres in the region distributed into three subregions (mountain, land and coast). Three PHC centres from each subregion were selected for acquiring the sample. Participants were recruited while visiting the selected PHC centres. Face-to-face interviews were conducted via trained medical students.

### Inclusion criteria

All adult citizens (age ≥18 years) residing in the Jazan Region of Saudi Arabia of both sexes and who had agreed to participate were included and considered appropriate for the study.

### Exclusion criteria

Citizens <18 years and unwilling to participate were excluded. Those extremely ill or unable to communicate were also excluded from the study.

### Data collection tools

Given the unavailability of a standard questionnaire, the authors in a previous study conducted in Riyadh of Saudi Arabia in 2014, developed a self-reported questionnaire to focus on awareness of dehydration definition, symptoms, causes, prevention, water intake recommendation and fluid intake practices^(13)^. The questionnaire was drafted in English and translated into Arabic and back-translated to validate the accuracy of the translation prior to validation. The final questionnaire was distributed to 393 participants and their results were published in 2018. Permission for use was taken from the authors and Arabic version was received. The first page of the questionnaire comprised clear information about the study's objectives and clarified information about participation, confidentiality, withdrawal and informed consent. The questionnaire consisted of twenty-six questions and comprised four primary sections: the first gathered information on the respondents’ socio-demographic and disease characteristics, including age, sex, nationality, education level, work status, monthly income, history of chronic diseases and health-related behaviours; the second assessed the participants’ knowledge of dehydration, including definition, clinical symptoms, consequences and treatment; the third section considered information about recommended daily water intake and the fourth sections assessed preventive measures taken to avoid dehydration.

The knowledge of dehydration symptoms, causes and consequences were based on dichotomy ‘yes’ and ‘no’ questions. Dehydration definition was assessed based on three options (I'll be dehydrated if I don't drink enough fluid, I'll be dehydrated if I don't eat properly and I'll be dehydrated if I don't have enough sleep). Knowledge was considered good when the participants selected the first or the second or all three options and considered poor when ‘no’ was selected the first option.

Dehydration prevention was assessed based on three preferences (dehydration risk can be reduced if I drink enough fluid, by consuming food with high water content, in hot weather fluid replenishment as priority). Good knowledge of dehydration preventions was considered when all or two preferences were selected ‘yes’. Knowledge was considered poor when the first preference was considered ‘no’.

The total water intake was assessed based on the number of water glasses used per day. Good knowledge of daily water intake recommendations was considered when the participants selected 2 or 3 l out of options.

### Data presentation and statistical analysis

The survey data were analysed using Statistical Package for the Social Sciences (SPSS) version 25. Frequencies and percentages were used for analysing the selected socio-demographic data. Poisson regression was used to determine the predictors of water cups consumptions, the scale parameter was set to Pearson *χ*^2^ to make the model fit the over dispersion in data. An independent *T*-test was used to check if there is a significant difference in the total fluid intake between hospitalised due to dehydration and those who were not. Results were reported as a 95 % confidence interval, a rate ratio and a *P* value. A *P* value less than 0⋅05 was used as the cut-off level for statistical significance.

## Results

### Socio-demographic and disease characteristics

A total of 440 participants filled the survey, 228 (51⋅82 %) were male, 212 (48⋅18 %) were female and the mean age of participants was 32⋅19 + 10⋅17 (Table 1). Out of the total, 406 (92⋅27 %) were Saudi. The mean BMI of participants was 25⋅28 + 5⋅29. Of the participants, 249 (56⋅59 %) had received higher education and 144 (32⋅70 %) were professionals. With regard to health and health-related behaviours, 56 (12⋅70 %) reported diabetes, 42 (9⋅50 %) reported hypertension and 82 (18⋅60 %) were smokers (Table 1).

**Table 1.** Socio-demographic and diseases profiles of the participants (*n* = 440)

Demographic	Number (*N*)	Percentage (%)
Age (mean, sd)	32⋅19	10⋅17
BMI (mean, sd)	25⋅28	5⋅29
Gender (*n*, %)		
Male	228	51⋅82
Female	212	48⋅18
Nationality (*n*, %)		
Saudi	406	92⋅27
Non-Saudi	34	7⋅73
Educational level		
Primary	17	3⋅86
Secondary	27	6⋅14
Diploma	147	33⋅41
University	249	56⋅59
Occupation (*n*, %)		
Professionals	206	46⋅8
Workers	86	19⋅5
Housewives	60	13⋅6
Students	76	17⋅3
Monthly income SR (*n*, %)		
<3000	199	45⋅2
3000–4900	53	12
5000–8999	63	14⋅3
9000–14 999	93	21⋅1
>15 000	32	7⋅3
Reported chronic diseases		
Diabetes	56	12⋅7
Hypertension	42	9⋅5
Heart diseases	9	2⋅0
Kidney diseases	25	5⋅7
Health-related behaviours		
Smoking	82	18⋅6
Khat chewing	46	10⋅5

### Knowledge of dehydration definition, prevention, consequences and water intake recommendation

Good knowledge of dehydration definition, dehydration prevention and recommended minimum water intake was observed in 431 (97⋅95 %), 417 (94⋅8 %) and 330 (75 %) of the participants, respectively (Table 2). Regarding dehydration consequences, 331 (75⋅2 %) reported that severe dehydration can could to kidney renal stones, 113 (25⋅7 %) reported that severe dehydration could lead to brain damage and 76 (17⋅3 %) reported that severe dehydration could lead to seizers.

**Table 2.** Knowledge of dehydration definition, prevention, consequences and water intake recommendation

Question	Selected (*n*/%)	Not Selected (*n*/%)
Knowledge of dehydration definition		
I can become dehydrated if I don't drink enough fluids	410 (93⋅2 %)	30 (6⋅8 %)
I can become dehydrated if I don't eat properly	21 (4⋅80 %)	419 (95⋅2 %)
I can become dehydrated if I don't get enough sleep	8 (1⋅81 %)	432 (98⋅19 %)
I don't know	1 (0⋅23 %)	439 (99⋅77 %)
Overall knowledge	Good 431 (97⋅95 %)	Poor 9 (2⋅05 %)
Knowledge of dehydration prevention		
By drinking enough fluids (water/milk/juice/tea)	417 (94⋅8 %)	23 (5⋅2 %)
By consuming foods with high water content (e.g. watermelon, oranges, apples, etc.)	355 (80⋅68 %)	85 (19⋅31 %)
In hot climate, replenish fluids as priority	355 (80⋅68 %)	85 (19⋅31 %)
Overall knowledge	Good 417 (94⋅8 %)	Poor 23 (5⋅2 %)
Knowledge of dehydration consequences		
Kidney stones	331 (75⋅2 %)	109 (24⋅8 %)
Death	177 (40⋅2 %)	263 (59⋅8 %)
Brain damage	113 (25⋅7 %)	327 (74⋅3 %)
Seizure	76 (17⋅3 %)	364 (82⋅7 %)
Knowledge of water intake recommendation		
1 l	41 (9⋅3 %)	399 (90⋅7 %)
2 l	133 (30⋅2 %)	307 (69⋅8 %)
3 l	197 (44⋅8 %)	243 (55⋅2 %)
4 l	69 (15⋅7 %)	371 (84⋅3 %)
Overall knowledge	Good 330 (75 %)	Poor 110 (25 %)

### Commonly reported causes of dehydration

**Fig. 1.**
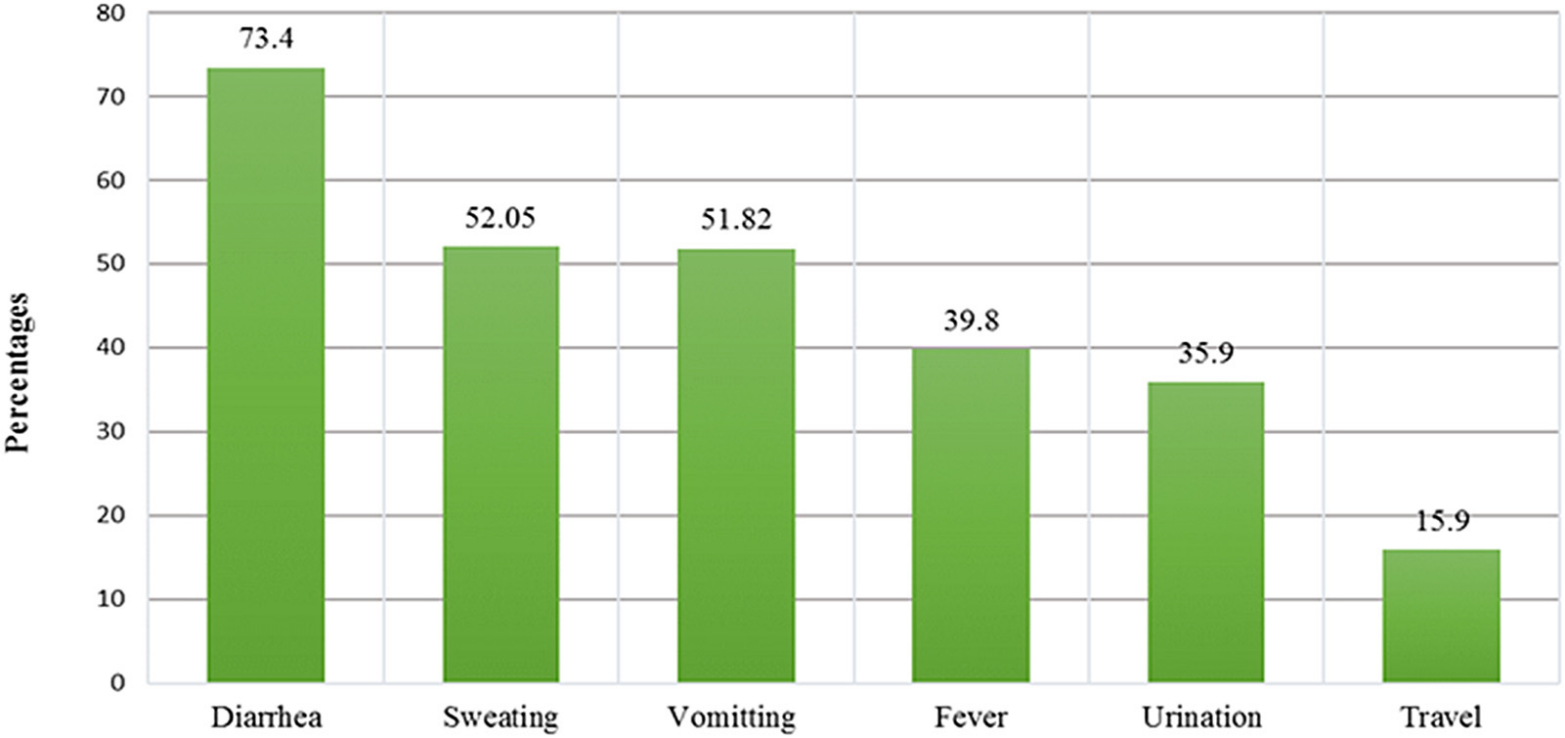
Reported dehydration causes.

The commonly reported causes of dehydration were diarrhoea (73⋅4 %), sweating (52⋅05) and vomiting (51⋅82). Fever (39⋅8 %), increased urination (35⋅9 %) and flight travel (15⋅9 %) were less reported causes of dehydration (Fig. 1).

### Commonly reported factors that affect restoring fluid loss

**Fig. 2.**
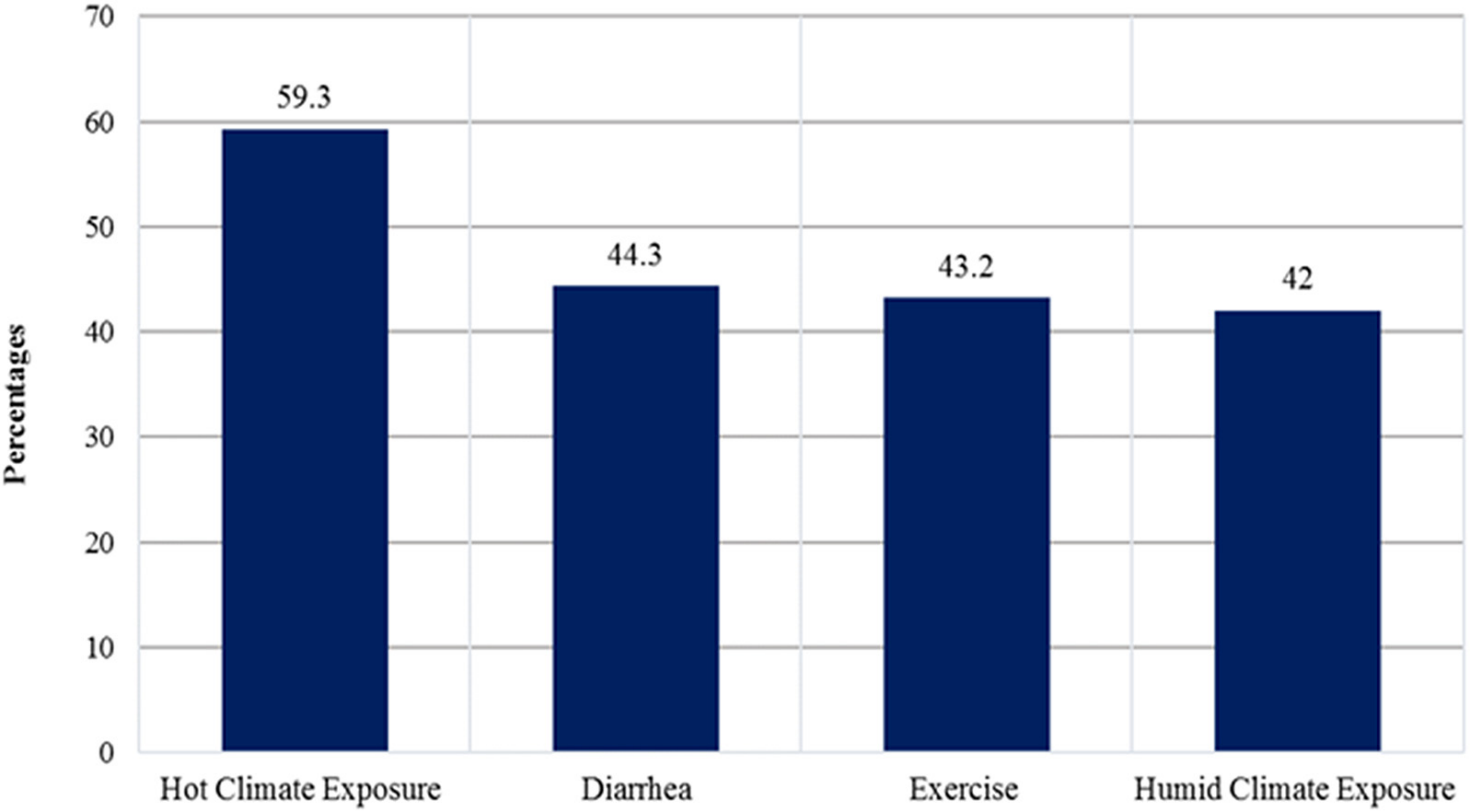
Reported factors that affect restoring fluid loss.

Hot climate exposure (59⋅3 %), diarrhoea (44⋅3 %) and exercise (43⋅2 %) were the commonly reported factors to affect restoring fluid loss (Fig. 2).

### Commonly reported symptoms of dehydration

**Fig. 3.**
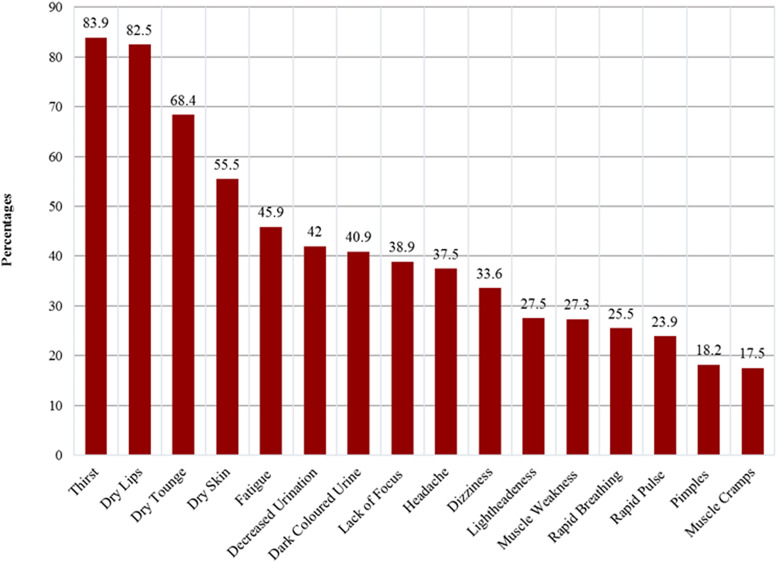
Reported symptoms of dehydration.

Thirst (83⋅9 %), dry lips (82⋅5 %) and dry skin (55⋅5 %) were the most frequently reported symptoms, while dizziness (33⋅6 %), lightheadedness (27⋅5 %) and muscle cramps (17⋅5 %) were the less frequently reported symptoms (Fig. 3).

### Predictors of water intake

More water intake was observed by increment in age, being diabetic and with prior hospitalisation due to dehydration (*P* value = 0⋅006, 0⋅023 and 0⋅037, respectively). The participants were reported more water intake with intake of additional glasses of coffee (*P* value = 0⋅004) and juice (*P* value < 0⋅001). More water intake was not significantly affected by gender differences, educational level and BMI status (*P* value = 0⋅24, 0⋅278 and 0⋅142, respectively; Table 3).

**Table 3.** Predictors of water intake

Predictors	Incidence Rate Ratio	95 % confidence limits	*P* value*
Intercept	1⋅376	1⋅025, 1⋅846	0⋅034*
Age	1⋅01	1⋅003, 1⋅017	0⋅006*
Gender (Male *v*. Female)	0⋅24	0⋅950, 1⋅227	0⋅24
BMI Category (Normal *v*. Obese)	1⋅141	0⋅957, 1⋅360	0⋅142
BMI Category (Overweight *v*. Obese)	1⋅101	0⋅831, 1⋅458	0⋅504
BMI Category (Underweight *v*. Obese)	1⋅005	0⋅840, 1⋅204	0⋅953
Education Level (Primary *v*. University)	0⋅836	0⋅604, 1⋅156	0⋅278
Education Level (Diploma *v*. University)	1⋅128	0⋅884, 1⋅439	0⋅332
Education Level (Secondary *v*. University)	0⋅987	0⋅865, 1⋅126	0⋅848
History of diabetes mellitus (Yes *v*. No)	1⋅225	1⋅028, 1⋅459	0⋅023*
History of Hypertension (Yes *v*. No)	1⋅007	0⋅815, 1⋅244	0⋅949
History of Heart Disease (Yes *v*. No)	0⋅637	0⋅387, 1⋅047	0⋅075
History of Kidney Disease (Yes *v*. No)	1⋅093	0⋅854, 1⋅398	0⋅48
History of Hospitalisation due to Dehydration (Yes *v*. No)	1⋅18	1⋅010, 1⋅378	0⋅037*
Coffee Intake	1⋅067	1⋅02, 1⋅115	0⋅004*
Juice Intake	1⋅068	1⋅039, 1⋅098	<0⋅001*
Tea Intake	1⋅005	0⋅966, 1⋅047	0⋅793
Soda Intake	0⋅956	0⋅912, 1⋅002	0⋅059

* *P* value is based on the Poisson regression model (less than 0⋅05 was used as the cut-off level for statistical significance).

## Discussion

The aim of the present study was to determine the awareness of dehydration state and fluid intake practice in the Jazan Region of Saudi Arabia. Poor awareness of dehydration and fluid intake was reported in different countries worldwide^(10,11)^. Nearly, 50 % of the adult population fail to run across the recommended total water intake despite the adverse health outcomes associated with chronic low fluid intake^(15)^. The participants in the present study reported good knowledge of dehydration definition and fluid intake practice. One-third of them did not meet the required minimum water intake and they have poor knowledge of dehydration consequences and some of dehydration symptoms. Focus education directed to the dehydration symptoms and complications will help to improve the overall hydration state.

Though the participants showed good knowledge levels with regard to the common symptoms of dehydration like excessive thirst, dry lips and dry skin and were able to elicit the common causes of dehydration like diarrhoea, sweating and vomiting, their knowledge regarding headache, dizziness and muscle cramps, as symptoms and fever and excessive urination, as causes of dehydration were poor. These variations in knowledge assessment were similar to the results of the cross-sectional survey of knowledge assessment of dehydration among people living in Riyadh City during summer^(13)^.

In the present study, the participants were aware of drinking water, as almost two-thirds of them agreed to drink 3 l or per day, which is consistent with institute of medicine (IOM) guidelines and with the results of a survey conducted among school students in China^(16)^. Based on the recommendations of IOM, water intake of ≥3⋅7 l daily was considered adequate for men and ≥2⋅7 l daily was considered adequate for women^(17)^. In the arid climate, more water intake is needed and 4⋅1–6⋅0 l is the minimum recommended daily intake for a healthy, 70 kg adult^(18)^. Good awareness of water intake consistent with good knowledge regarding dehydration definition was similarly reported by Shaheen *et al.* in Saudi Arabia^(13)^. Adequate water intake is very important in hot climate and in sport. To prevent dehydration, water intake should be replenished regularly^(19)^.

Regarding knowledge of dehydration consequences, three-fourth of the participants in the present study reported that severe dehydration could lead to the development of renal stones. However, less than one-third of the participants agreed that severe dehydration could lead to seizures, brain damage and even death. The risk of developing renal stones and urinary tract infections due to dehydration is well observed in the literature^(20–22)^. The impact of chronic dehydration could extent to chronic kidney diseases (CKD) especially in the present of co-morbid diseases and extreme hot climate^(23)^. Limited knowledge of dehydration consequences were reported by Zuo *et al.*, in China, and Shaheen *et al.*, in Saudi Arabia^(11,13)^. The impact of severe dehydration on brain functioning has been well established in the literature, particularly in elderly adults and young children^(24–27)^. Even mild dehydration has been linked to significant effects on everyday functioning^(28)^. Thus the beneficial hydration effect was not only observed for speed of cognitive functioning but also for processing of everyday functioning.

Regarding the predictors of water intake, increment in age, coffee intake and juice intake were associated with more water intake. Similarly, juice intake has been reported as a predictor of water intake by Shaheen *et al.*^(13)^, while coffee intake has been reported as a predictor of water intake by Christopher among university students^(29)^.

Theoretically, when the people were well educated, their knowledge of dehydration is expected to be increased and their intake of hydration recommendations will be better than less educated people. However, in the present study, although the majority of the participants received high education, we did not find a significant correlation between hydration knowledge and the level of education. Same finding was reported by Shaheen *et al.*, in Riyadh City. Large sample size cross-sectional and more qualitative studies are needed to test this correlation.

Weight status has not been associated with more water intake in this study, while it was reported as a predictor of more water intake in the literature^(13,30)^. In contrast to the present study, Shaheen *et al.* and Goodman *et al.* had reported participants aged 55 years and above drink less compared with the younger group. Additional glass of tea was not found to be linked with more water intake, while it was reported as a predictor of more water intake by Shaheen *et al.* Being diabetic and prior history of hospitalisation were found to be linked with more water intake in the present study, while contradicting responses were observed by others^(13,30)^. These variations in the predictors of water intake could be explained by human nature and different perceptions. Variation in sample size and assessment technique could also explain disparities in the predictors of water intake in different populations.

The results of the present study provide fresh knowledge and reflect the state of water intake in the Jazan Region. The sample was taken from the randomly selected PHC centres, which nearly represented the population under the study. However, being cross sectional will limit the generalisation of the results. A small sample size is also one of the limitations. A large sample size study will help to test the correlation between the different predictors and water intake practice.

## Conclusion

The participants exhibited good knowledge of dehydration definition and recommended minimum water intake. However, the knowledge was lacking for the less common symptoms, causes, and of potentially serious consequences of dehydration. Intake of fluids such as ‘juice and coffee’ enhances more water intake. Although two-thirds of the participants met the recommended daily water intake, still one-third of them did not meet this level.

Innovative approaches to enhance healthy drinking are warranted and may include partnering with patients to take an active role in hydration monitoring and increasing communication with the different healthcare providers.
